# Implementation of observational pain management protocol to improve pain management for long-term institutionalized older care residents with dementia: study protocol for a cluster-randomized controlled trial

**DOI:** 10.1186/1745-6215-15-78

**Published:** 2014-03-13

**Authors:** Justina Yat Wa Liu, Claudia Lai

**Affiliations:** 1 Centre for Gerontological Nursing, School of Nursing, The Hong Kong Polytechnic University, Hung Hom, Hong Kong

**Keywords:** Pain management, Pain measurement, Dementia care, Nursing homes

## Abstract

**Background:**

Systematic use of observational pain tools has been advocated as a means to improve pain management for care home residents with dementia. Pain experts suggest that any observational tool should be used as part of a comprehensive pain management protocol, which should include score interpretation and verification with appropriately suggested treatments. The Observational Pain Management Protocol (Protocol) was therefore developed. This study aims to investigate the extent to which the implementation of this Protocol can improve pain management in care home residents with dementia.

**Methods/design:**

In this two-group, single-blinded, cluster-randomized controlled trial, 122 care home residents with dementia and pain-related diagnoses will be recruited from eight care homes (that is 15 to 16 residents from each care home). Invitations will be sent to all local care homes who meet the home selection criteria. The eight care homes will be randomly selected from all care homes that agree to join this trial. They will then be randomized to either the control or experimental conditions. Participants from each care home will be placed into their home’s corresponding group to avoid ‘contamination’ effects across participants. Each intervention cycle will take 16 weeks (that is, baseline assessment and care home staff training for 4 weeks and Protocol implementation for 12 weeks). The Protocol will guide the pain management of the participants in the experimental care homes. Meanwhile, the control care homes will continue their usual pain management strategies. Intervention effects will be measured weekly during the protocol implementation period and compared with the baseline measurements, as well as between the experimental and control conditions.

**Discussion:**

Although similar pain protocols have been suggested previously, the recommendations were based on experts’ opinions rather than evaluation of research studies. The feasibility and effectiveness of this kind of pain management protocol, tailored to older people with dementia, remains unknown. The findings of this trial will offer strong evidence that better strategies for pain management should be used in the care home daily routine.

**Trial registration:**

The Chinese University of Hong Kong, Centre for Clinical Trials: CUHK-CCT00367

## Background

Despite a high prevalence of pain among older care home residents, pain management for this group remains suboptimal, especially for those with dementia. Among long-term care home residents with dementia, pain often goes unrecognized and under-treated. Evidence shows that these people receive considerably lower doses of analgesics than their cognitively intact counterparts [1]. The discrepancy between the prescribed and actual doses widens as cognition decreases [2]. Residents with dementia are prescribed and administered significantly fewer pain medications and in smaller doses than those without dementia [3,4]. Evidence shows that the provision of inadequate pain treatment for residents with dementia is partly due to inadequate pain assessment and documentation [5].

Lack of a standardized pain assessment tool and proper documentation are major barriers to successful pain management for residents with dementia [5]. Poor pain management in this population has been partly attributed to difficulties with efficient pain assessment. As the deterioration of cognition affects individuals’ ability to report pain, there is an increasing risk that pain will go undetected, leading to poor pain management. Unrelieved pain in residents with dementia may lead to fear of movement, resistance to care, anxiety, and agitated behavior [6]. Untreated pain not only affects their quality of life, but also causes more stress to caregivers dealing with problematic behavior.

During the last decade, much effort has been made to improve pain management for older people who cannot verbalize their pain. Pain experts have suggested strategies for better pain management for them by using observational pain tools, setting standardized pain management strategies, and through education and training [7,8]. Additionally, at least 15 observational pain tools have been developed and validated during the last decade. Generally, preliminary data support the psychometric properties of several observational pain tools [9-12]. The systematic use of an observational pain scale has been advocated as a means to improve pain management [7,8], but so far only one experimental study has been conducted to evaluate the effect of an observational pain tool on pain management. Fuchs-Lacelle et al.’s study demonstrated that regular use of an observational pain scale for 3 months in long-term care homes improved pain management, as evidenced by increased use of analgesics when compared with the control group [13]. However, this study only evaluated a single observational pain tool’s effect in improving pain management practice.

Scale application and score interpretation were considered major challenges to the adoption of an observational pain tool in daily clinical routines. Additionally, none of the behavioral indicators (such as grimacing, fidgeting, crying, shouting, and so on) used in the observational pain tools are unique to pain. There was a concern that misinterpretation of residents’ behavior may lead to inappropriate treatment [14]. Several researchers suggested that observational pain scores should not be considered definitive. Rather, any observational pain tool should be used as part of a comprehensive pain management protocol. All strategies suggested for improving pain assessment and management for people with dementia should be incorporated in the protocol [15-17]. For instance, multiple levels of assessment and treatment should be included in the protocol. In addition to observing residents’ pain-related behavior using an observational pain tool, investigating possible causes of pain, getting information from surrogates, and attempting to obtain self-reported pain should be undertaken for preliminary verification that the observed behavior is pain-related. Observational pain score interpretation should be suggested and pain treatments should be recommended according to the pain scores. Additionally, the protocol should suggest subsequent actions if the behavior persists. This protocol should be used daily. All assessments and treatments should be documented to allow continuous monitoring. However, these suggestions appeared mainly as expert consensus statements [7] or recommendations [15], rather than empirical evidence obtained based on scientific research studies.

In view of this, a comprehensive Observational Pain Management Protocol (Protocol) has been developed and pilot-tested by the first author (JL) in a long-term care home [18]. The flow of this protocol is guided by the pain management process and the corresponding actions (Table 1). The process includes observing and documenting participants’ pain-related behavior using a standard observational pain assessment tool (the Chinese - Pain Assessment IN Advanced Dementia [C-PAINAD]). Other steps include investigating possible causes of pain, getting information from surrogates, attempting to obtain self-reported pain, initiating different types of pain treatment according to observed pain scores, and performing re-assessment after pain treatment.

**Table 1:** Observational pain management protocol

**Process**		**Actions**
1	Pain assessment	Using C-PAINAD for pain assessment
*2a	Score verification	Investigating possible causes of pain (such as injury or pain-related diagnosis), obtaining self-reports if at all possible by asking simple yes/no pain questions to participants, getting information from surrogates, direct contact nurses, and so on
3	Score interpretation	0-1 = no pain; 2-3 = mild pain; ≥ 4 = moderate pain or above
4	Pain-relieving interventions	**Stage one (Pain score > 1)**:
		Initiating pain-minimizing and caregiving guidelines^a^
		**Stage two (Pain score > 4)**:
		Non-pharmacological treatments: hot therapy, cold therapy, TENS, massage, and so on
		- Consulting in-house physiotherapists, occupational therapists and nurses about the selection of treatment(s)
		- Pharmacological treatments: analgesic trial
		- Administering regular/‘if needed’ (PRN) analgesic medications 30 minutes before pain-triggered nursing procedures
		- When no analgesic has been prescribed, discussing with resident’s physician whether or not to prescribe analgesics
5	Evaluation and continued monitoring	Monitoring the effectiveness of the implemented interventions by C-PAINAD
		- Decreased pain score - continued monitoring
		- If pain-related behavior persists - modify interventions
6	Documentation	All pain scores and pain treatments administered to participants must be recorded on the pain chart
2b	*Verification - no evidence indicates pain	- Attempting to interpret meaning of behavior with help of caregivers who are familiar with the residents
		- Ensuring basic needs are met

^a^Please refer to Table 2 for detailed descriptions of the Pain-minimizing and Caregiving Guidelines.

The Protocol was used to guide the pain management of 30 care home residents with dementia, with at least one pain-related diagnosis, for 8 weeks. Before the implementation, only five residents had been prescribed pain medications. Pain medications administered to the residents were quantified by the Medication Quantification Scale (MQS) [19]. During the study period, the mean MQS score increased from 10 (SD 14.23) at week 1 to 23.41 (SD 10.58) at week 8. The percentage of residents given pain medications increased to 23.33% (n = 7) at week 8. Similarly, only six residents were receiving non-pharmacological interventions at week 1. Towards the end of the study period, 17 residents were receiving non-pharmacological pain-relieving interventions such as hot pads, ice therapy, and massage. The mean pain score of the 30 residents decreased slightly from 2.87 (SD 1.30) at week 1 to 2.57 (SD 1.32) at week 8. Although this study did not show a statistically significant reduction of pain scores, probably due to the small sample size, it did show clinical relevance in improving pain management. Incorporation of this Protocol into the long-term care home’s daily routine encouraged well-organized pain recording for each resident, allowing further comparisons of pain treatment effectiveness. Besides this pilot study, no other study has been conducted to evaluate the effectiveness of similar protocols in improving pain management practice.

The aim of this study is to investigate the extent to which the implementation of the Observational Pain Management Protocol can improve the management of pain for older care home residents with dementia. Based on the findings in our pilot study [18], we hypothesize that the implementation of the Protocol will result in an improvement in pain management for residents with dementia, as manifested in an increased use of both pharmacological and non-pharmacological pain treatments. As a consequence, behavioral pain scores will be lower due to better pain management. The following two research objectives have been derived:

1. To investigate whether a systematic use of the Protocol improves pain management, as manifested by the increased use of pharmacological and non-pharmacological pain treatments for residents with dementia.

2. To investigate whether the use of this Protocol results in lower observational pain scores for residents with dementia.

## Methods/design

### Study design

**Figure 1:**
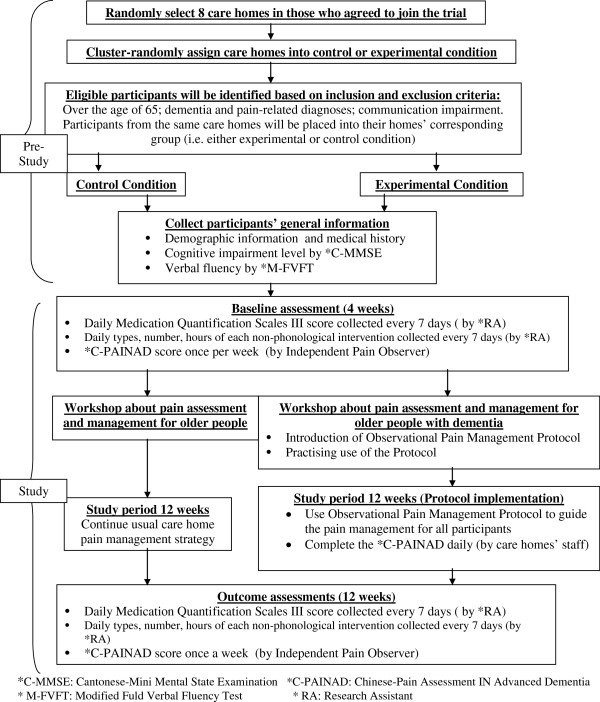
Trial design.

A two-group, single-blinded, cluster-randomized controlled trial (RCT) will be used to examine the effect of the Protocol on pain management for older people with dementia as compared to a control condition of usual pain management practice in long-term care homes. This study will take 16 weeks (that is, baseline assessment and care home staff training for 4 weeks and Protocol implementation for 12 weeks). Participants in the control condition will continue usual pain management practice according to their care homes. Intervention effects will be measured weekly and compared with the weekly baseline measurements for 4 weeks, as well as between the experimental and control conditions, to detect the progress of the effectiveness of the Protocol in pain management continually throughout the study period. The overall study design is illustrated in Figure 1.

### Study settings and participants

This study will be conducted in long-term care homes for older people who suffer from physical and/or mental disabilities with deficiencies in coping with daily life.

#### *Inclusion criteria for long-term care homes*

The long-term care homes, funded by the government, must be under the Social and Welfare Department of Hong Kong (HK) and thus meet a set of standard criteria as set by the HK Government. The staffing pattern, staff-resident ratio, bed space, the other service criteria of the facilities, and the nature of the residents are therefore similar in that they have to meet government standards. Additionally, the total number of residents must be 100 or above so as to maximize the chance of recruiting a sufficient number of participants in each care home (that is 15 to 16 - please refer to sample size for details).

#### *Exclusion criteria for long-term care homes*

Private nursing homes will be excluded due to the variations in their staffing pattern, the staff-resident ratio, bed space, the other service criteria of the facilities, and the nature of the residents.

The target population of this study is long-term care residents with dementia, communication impairment, and pain problems.

#### *Inclusion criteria for participants*

Participants must be ≥65 years old, of either gender. They must have been living in the long-term care home for >6 months to avoid anxiety and distress due to the unfamiliar new environment. Their general health must be stable (with no acute physiological or psychiatric illnesses, and no history of fractures or emergency hospital admissions within the past 3 months). Participants must be officially diagnosed with some form of dementia according to the DSM-IV criteria for dementia. Their cognitive state will be assessed by the Cantonese-Mini Mental State Examination (C-MMSE) [20]. The participants must have at least one pain-related diagnosis such as osteoarthritis, low back pain, or rheumatoid arthritis. Each participant’s communication ability will be assessed by the communication score based on two items in the Hong Kong version of the interRAI-Home Care (HC) Assessment (formerly called the Minimum Data Set-HC): (1) making one’s self understood; and (2) ability to understand others. These two items are rated on a 5-point scale: 0 = understood, 1 = usually understood, 2 = often understood, 3 = sometimes understood, 4 = rarely or never understood. A total communication score is summed from these two items and ranges from 0 to 8, with 0 meaning no communication problems and 8 meaning very severe communication impairment. Participants must have a communication score ≥5 to be eligible for recruitment into this study [21].

#### *Exclusion criteria for participants*

Exclusion criteria includes residents with: (1) a current medical condition and the need to be frequently admitted to hospital during the study period (for example, acute exacerbation of chronic obstructive pulmonary disease or renal failure, and so on); (2) co-morbid psychiatric disorders (for example, psychosis); or (3) a recent experience of distressing social circumstances (for example, the death of a close relative or friend within the previous 6 months) that may cause altered behavior patterns; and (4) terminal illness (for example, advanced cancer) and deemed not to have more than 6 months to live.

Although many kinds of chronic diseases such as diabetes mellitus and ischemic diseases may affect the pain sensation of older people, it is not uncommon for them to have multiple health problems. Residents who match all the inclusion criteria but have other kinds of chronic illnesses will still be recruited, providing the illnesses are not acute. Each participant’s medical history will be recorded in detail.

#### *Sample size*

We will randomly select eight long-term care homes that agree to join this study. As no similar cluster-randomized trial has been reported in the literature, we can only estimate the intracluster coefficient with a range between 0 and 0.1 [22]. The intensity of pain can vary according to the different natures and sources of the pain. As this study is not limited to participants suffering from a particular type of pain, we anticipate that variations in the nature of participants’ pain will be considerable. Due to its heterogeneous nature, we assumed an intracluster correlation of 0.02. The pilot study was able to detect a mean difference of 13.41 points on the MSQ score (95% confidence interval 8.923 to 17.897) and -0.3 points on the C-PAINAD (95% confidence interval -4.459 to 0.169) between the pre- and post-tests [18]. After averaging the effect sizes calculated based on these two mean differences, this was equivalent to an effect size of 0.65. In order to secure an appropriate effect size, a more conservative approach of using an effect size of 0.4 was used to calculate the sample size. With a significance level (α) of 0.05 and power (1-β) 0.8 for a two-sided test, the sample size calculation gives about 38 participants for each group in order to evaluate a mean difference in MSQ and C-PAINAD scores between the experimental and control groups. Accounting for the intracluster correlation, a variance inflation factor of 1.31 will be multiplied to this calculated sample size. Further accounting for a 15% drop-out rate over the period based on two similar studies [13,18], a total number of 61 participants per group is required, resulting in a total proposed sample size of 122. This means we will recruit 15 to 16 participants in each long-term care home.

### Randomization and allocation concealment

The care home list is generated from the website of the Social and Welfare Department of HK, which includes all local government-funded long-term care homes. An invitation letter has been sent to 75 local long-term care homes that meet the home selection criteria. Based on our previous experience, the response rate will be around 15% to 20%. This means we anticipate that about 11 to 15 care homes will accept our invitation. A random list and a replacement list will be generated. Eight care homes will be randomly selected from all care homes who accept the invitation.

The homes will then be randomized to the control or experimental condition according to the pre-set computer-generated randomization list generalized by a biostatistician who will not be involved in this study. Random group allocations will be sealed in opaque envelopes. Each care home will be viewed as one unit throughout the entire study. As mentioned in the section on home selection criteria, the settings of the home should be similar, and we will not stratify or match the homes during the randomization process. Random allocation will be performed after receiving the homes’ indication of their willingness to join the study. We anticipate that this recruitment process will take about 3 to 4 months. The researcher will then open the random allocation envelopes in order and allocate nursing homes to either the experimental or control condition accordingly. Participants from each long-term care home will be placed into their care homes’ corresponding group to avoid ‘contamination’ effects across participants within the experimental and control conditions. Participant recruitment will be conducted after the care homes have been randomly allocated to either group. Each care home will recruit 15 to 16 participants. We anticipate that it will take about 3 to 4 weeks to complete participant recruitment in each nursing home. The study, including staff training, baseline assessment, and protocol implementation, will start after we have recruited sufficient numbers of participants in each nursing home.

### Intervention

Residents who meet the sample selection criteria will be recruited. We will interview potential participants and their relatives to explain the purpose of the project. Their cognitive level will be assessed with the C-MMSE, whereas their communication ability will be assessed by two items in the Hong Kong version of the interRAI-HC assessment during participant recruitment. Additionally, their verbal fluency will be assessed by the Modified Fuld Verbal Fluency Test (M-FVFT) [23]. Demographic data, pain-related diagnoses, and medical history will be collected by reviewing participants’ medical records.

#### *Baseline assessment*

Baseline assessment will include the weekly MQS III score and the weekly record of non-pharmacological pain treatments. Participants’ daily use of pain medications will be quantified into the MQS III scores. Other non-pharmacological pain-relieving interventions will also be recorded daily. Both of these records will be collected weekly for the first 4 weeks of the intervention cycle by the designated research assistant (RA). This collection will continue for the 12 weeks of the Protocol implementation in the experimental care homes or during the usual pain management period in the control care homes. In addition, participants’ C-PAINAD scores will be collected by the independent pain observer once a week throughout the 16-week study period. Please refer to the section below for a detailed explanation of the role of the independent pain observer.

#### *Implementation of the Protocol in experimental long-term care homes*

Four 1-hour workshops will be conducted by JL during the first 4 weeks of the intervention cycle. These will aim to ensure that both professional and non-professional care home staff understand and follow the Protocol correctly. Workshop I will introduce pain assessment and management for older people with dementia. Workshops II and III will explain the use of the Protocol for their pain assessment and treatment. The staff will practise pain assessment by C-PAINAD through various pre-developed clinical vignettes. They will also learn to discriminate between various steps of pain treatments based on the pain score. Group discussions will be held after the practice. The staff will be asked to explain their choice of indicators and give reasons for their choice. They will also be asked to explain their subsequent actions in terms of the selection of different stages of pain treatments suggested by the Protocol. Through practice and discussion sessions, the staff should be able to follow the Protocol. Workshop IV is mainly about the implementation of the Protocol. Finally, a test will be conducted to ensure that the staff understand the Protocol. Given that the job nature and educational backgrounds of professional and non-professional caregivers are different, they will attend the workshops separately. The workshops’ contents will be tailored to suit these two groups of staff.

Following the training workshops and after ensuring that all care home staff understand and can follow the Protocol (Table 1), it will be formally incorporated into the care homes’ daily routine.

1. *Pain assessment*: The staff will perform a daily observational pain assessment using the C-PAINAD when participants are undergoing potentially pain-triggering nursing procedures or exercising. Since pain-related behaviors are more likely to be observed when pain is triggered by movement [24], pain may also be exacerbated by the movement that occurs with different types of nursing activities [25-27]. This means that nursing care can potentially trigger pain. Studies have shown that the PAINAD is more sensitive in detecting pain while patients are moving or exercising [28]. The pain assessment suggested in this protocol will be used at least once per day when participants are undergoing potential pain-triggering nursing procedures or exercising. Since most of these nursing procedures are scheduled in the morning shift, the staff will be instructed to perform observational pain assessment at least once per day during the AM shift. They will also be encouraged to re-assess participants’ C-PAINAD scores whenever necessary.

2. *Pain score interpretation and verification*: On the C-PAINAD scale, 0 indicates no pain and 10 indicates severe pain. In this study, C-PAINAD >1 will be considered an indication of pain or discomfort. Pain treatments should then be implemented. The PAINAD was converted into a categorical scale based on items in the 4-point Verbal Rating Scale, in which 0-1 corresponded to no pain; 2-3 to mild pain, and a score of 4 and above would correspond to moderate pain and above [29]. These findings will be used to interpret the C-PAINAD score. However, since none of the pain-related behaviors are specific to pain, verification procedures are needed. First, it is suggested that the staff investigate the possible causes of pain (for example, history of recent injury or pain-related diagnosis). Second, they need to obtain the participant’s self-report if at all possible, by asking simple yes / no pain questions to participants. Whenever necessary, the staff should also collect information from surrogates, direct contact nurses, and so on. If there is no evidence indicating pain, the staff should attempt to interpret the meaning of the behavior in question with the help of others who are familiar with the participant. Additionally, the staff are also required to ensure that participants’ basic needs are met (for example, check whether they are hungry, thirsty, or seeking attention for another reason). If evidence shows that the observed behaviors are highly likely to be caused by pain, staff are required to implement treatment to relieve pain.

3. *Implementation of pain-relieving interventions*: In many circumstances, pain can be alleviated by simply removing or correcting the cause, such as maintaining a proper body alignment while transferring residents with back problems [30]. The Pain-minimizing and Caregiving Guidelines designed by Talerico and her colleagues [31] (Table 2) were therefore adopted in this protocol and will be considered as the first step in pain treatment. The guidelines should be implemented when the C-PAINAD score >1. They aim to make caregivers aware of participants’ behavioral pain expressions during potential pain-triggering nursing procedures. Signs will be placed at bedsides to remind caregivers to take great care while conducting nursing activities. If the interventions in Step 1 fail to decrease a participant’s C-PAINAD scores, Step 2 pain treatment should be initiated. If the C-PAINAD score >4, Step 2 should also be implemented. Step 2 interventions involve both non-pharmacological and pharmacological treatments. The treatment choices at this stage are tailored to the needs of particular participants. In-house physiotherapists, occupational therapists, and nurses-in-charge will be consulted about non-pharmacological treatment. If residents have been prescribed PRN analgesic medications, those medications should be administered. If residents are prescribed regular analgesic medications, these should be administered 30 minutes before the potential pain-triggering nursing procedures. If residents have not been prescribed any analgesics, nurses are advised to discuss the prescription of suitable medications with physicians.

**Table 2:** **Approaches to reducing the pain related to caregiving**[31]**]**

**Timely warnings**	- Place grab bars, transfer poles, and bed canes to assist with transfer and aid in self-directed care.
- Before a potentially painful movement or activity, give a warning, such as ‘I’m going to move your feet and put on your socks. Are you ready?’	
	- Brace painful knees during transfers.
	- Use a non-skid mat at the bedside to prevent sliding during transfers.
- Allow the resident to prepare for the action or movement.	- If the patient appears to be in pain, assess the usual transfer method for alternative, more comfortable ways of transferring. For example, beginning with two people, try the ‘carry transfer’ technique or use a mechanical lift.
- Wait for the resident to give permission (if she or he is able) before beginning the task.	
	- Evaluate the possibility of raising low beds from the floor to reduce pain associated with transfers, using a winged mattress to reduce the risk of falling.
	- Request an occupational therapy consultation for individualized techniques for transfers from low beds.
**Movement in bed**	
- Do not pull on arms when rolling or moving a resident in bed. Instead, grasp shoulders and hips, using a ‘log-roll’ technique to keep the body in proper alignment.	
- Use draw sheets to roll the patient from side to side rather than pulling and pushing on various parts of the body.	
**Transferring**	**Seating and positioning**
- If a patient has insufficient upper-body strength, raise the head of the bed and help the patient onto her or his side before bringing her or him to a sitting position.	- Get an individualized wheelchair assessment from a physical or occupational therapist.
	- Ensure that footrests are fitted to the patient.
- Do not pull on the patient’s neck when moving or transferring.	- Pad areas of wheelchairs that cause pressure.
	- Evaluate comfort of wheelchair cushions; provide comfortable inserts.
- Allow a patient time at the edge of the bed to get her or his bearings before completing the transfer.	
	- Adjust tilt-in-space wheelchairs every 1 to 2 hours to relieve pressure and change position.
- Raise electric beds high enough that legs are bent at the knee at slightly more than 90° to assist patients in coming to a standing position.	
	- Provide a variety of seating options throughout the day; avoid using ‘geri-chairs’ which lack support and do not offer a functional position.
- Make sure the patient’s feet are touching the floor before transferring from bed to chair, to allow the patient to bear as much weight as possible.	

#### *Independent pain observer*

The staff in the experimental care homes will be aware of the pain treatment received by the participants. In view of the fact that possible expectancy bias may develop among staff in the experimental group as the study progress, the observational pain scale C-PAINAD will also be completed by an independent pain observer, who will be blinded to the study’s hypotheses, the allocation of participants to the experimental/control conditions, and their total pain treatment consumption. As mentioned, the pain assessment will be conducted weekly during the 16-week study period (baseline assessment for 4 weeks and Protocol implementation for 12 weeks) in both the control and experimental homes. Participants will perform the standardized exercise programme while the pain observer is assessing their pain. In previous studies, the standardized exercise programme successfully triggered pain-related behavioral responses [32,33]. The exercise programme will include a set of passive range of motion (ROM) exercises involving the movement of all major joints. Each ROM will be repeated 10 times or moved to the point of resistance and held for 10 seconds. The participants will then be asked to perform a set of active motions that include changing position from lying to sitting, from sitting to standing, and then from standing to walking. The design of this standardized exercise programme was based on a literature review and followed consultation with a physiotherapist and a nurse with long-term care experience. This exercise programme is similar to the daily stretching exercises performed by physiotherapists in care homes. It helps older people retain joint flexibility and avoid joint stiffness. It was specially designed and has been used to evaluate the psychometric properties of four observational pain scales, including PAINAD [34]. It is believed that if participants suffer from pain under routine nursing care activities, this exercise programme will generate a level of pain similar to that experienced by the participants. This exercise will be performed by another well-trained RA who will be blinded to the study hypothesis and group allocation.

#### *Control long-term care homes*

As in the experimental long-term care homes, four 1-hour workshops will be conducted by JL, addressing pain assessment and management for older people, during the first 4 weeks of the intervention cycle. The control care homes will continue their usual pain management strategies for the participants for the remaining 12 weeks of the intervention cycle. Similarly, participants’ weekly C-PAINAD scores will be collected by the independent pain observer throughout the 16-week study period. Their weekly MQS III score and weekly record of non-pharmacological pain treatments will be collected by another RA throughout the intervention cycle.

### Blinding

Two RAs, who will be blinded to the study hypothesis and group allocation, will be responsible for all subject assessment measures. One will be responsible for collecting MQS III scores and non-pharmacological pain treatment records. Another will work as the independent pain observer to collect participants’ observational pain scores. The pain observer will also be blinded to participants’ total pain medication consumption. We anticipate that after the training sessions, the staff in the care home will become more sensitive to residents’ behavioral patterns. To avoid a contamination effect caused by different time points in the Protocol implementation of different participants within the same care home, the pain management Protocol will be implemented for all participants in each care home simultaneously.

### Outcome measures

The effectiveness of the Protocol on pain management will be evaluated by: (1) the use of pharmacological pain treatments, measured by the Medication Quantification Scale III (MQS III); (2) the use of all non-pharmacological pain treatments recorded on the participants’ pain management record; and (3) pain-related behaviors exhibited by participants, which will be assessed by an observational pain scale (that is, C-PAINAD). The Protocol will be used to guide the pain management of the participants in the experimental condition for 12 weeks (that is, from the fifth to the sixteenth weeks of the intervention cycle). Intervention effects will be measured weekly and compared with the baseline measurements as well as between the experimental and control conditions to detect the progress of the effectiveness of the Protocol in pain management continually throughout the study period. Baseline measurements will be collected for 4 weeks (that is, between the first and fourth weeks of the intervention cycle).

#### *Medication quantification scale version III (MQS III)*

The MQS III will quantify the usage of pain medications [19,35]. The MQS I was used as an outcome indicator to demonstrate that regular use of an observational pain tool could significantly increase the total dosage of pain medications [13]. The MQS quantifies pain medications according to their daily administered dosage, pharmacological classification, and detriment weight (that is, the potential of each medication to produce side-effects) [19]. The concurrent validity of the MQS I was established by calculating the correlation coefficient (r = 0.755, *P* <0.01; two-tailed) between MQS I scores and the mean clinical judgment of pain management professionals [19]. The MQS III was updated in 2003 to modernize medication classifications and their detriment weights [36]. The MQS III has been validated and applied to the study of pain regimens in various pain conditions [37-39].

#### *Record of non-pharmacological pain treatments*

The common non-pharmacological interventions used for relieving pain among older people include, but are not limited to, heat and cold therapy, massage, transcutaneous electrical nerve stimulation (TENS), and acupuncture. Only interventions initiated by healthcare professionals will be recorded. The type and the total time per week of the treatments will be recorded during the study period. The non-pharmacological pain treatment will be recorded weekly for 4 weeks by the same RA, who is also responsible for collecting MQS III scores, according to participants’ nursing, physiotherapy or occupational therapy records prior to Protocol implementation (baseline period) and for the 12 weeks of Protocol implementation.

#### *Chinese - Pain Assessment IN Advanced Dementia (C-PAINAD)*

The C-PAINAD consists of five pain behaviors: breathing, negative vocalization, facial expressions, body language, and consolability. Each behavior is rated from 0 to 2 according to the severity of behavior exhibited, thus giving a total score of 0 to 10. Construct validity was supported by the C-PAINAD’s ability to differentiate pain from other pain-free conditions. It can detect pain among people with dementia. Exploratory factor analysis was used to evaluate the construct validity in which one major factor (that is, pain) was extracted and the percentage of variance was 51.2% [40]. The correlation coefficient was 0.68 when comparing the C-PAINAD with the Pain Visual Analog Scale. Generally, the findings were similar to those of the initial study validating the original PAINAD [28]. The results showed that the C-PAINAD was a simple, reliable, and effective pain assessment tool for cognitively impaired older people. A well-trained independent pain observer (that is, another research assistant who will not be involved in collecting data related to pain treatments) will use the C-PAINAD to observe participants’ pain scores weekly during the 4-week baseline assessment period. This will then continue weekly for 12 weeks during the Protocol implementation. Nurses in the experimental condition will also use the C-PAINAD to observe the pain-related behavior of participants daily for the 12 weeks of Protocol implementation. Pain scores collected by nurses will be compared to those collected by independent pain observers.

### Intervention fidelity and training of independent pain observer/research assistant

Across the intervention period, daily to weekly visits by research personnel will be arranged for each care home during the 12-week period. Particularly during the first 2 weeks of the Protocol implementation period, a member of the research team will visit the care homes daily to ensure the smooth implementation of the Protocol. If staff have difficulty following the Protocol, meetings will be arranged to answer their concerns.

An independent pain observer will be trained by JL on how to use the C-PAINAD. She will also practise pain assessment using the C-PAINAD with various clinical vignettes. On-site practice will also be arranged. An acceptable inter-rater reliability (ICC = 0.9) will be established by comparing the pain scores rated by the observers with those rated by JL, who is experienced in using the C-PAINAD. JL will also instruct and evaluate the use of all instruments by the RAs. Monthly quality control meetings will be arranged with all research personnel in this study. The inter-rater reliability between the pain observer and JL will be checked at least monthly throughout the entire study period.

### Statistical analysis and outcome measurement

Data will be analyzed using the Statistical Package for the Social Sciences (SPSS 19). Descriptive statistics will be generated for the demographic data. Normality assumptions for the variables will be checked. The paired t-test or Wilcoxon’s signed rank test will be used to examine any significant difference in the use of pain treatments and the change in the C-PAINAD score in the experimental group before and after implementation of the protocol. Student’s t-test or the Mann-Whitney U test will be used to examine any significant differences between the control and experimental groups in terms of the outcome variables. For categorical and dichotomous outcome variables, a χ2 test will be used to identify any significant differences between the groups. A *P* value ≤0.05 will be considered statistically significant. Mixed effect modelling (MEM) will be further used to take into account the intracluster correlation, to measure changes in the outcome measures after intervention with respect to its baseline, and to see the effectiveness of the intervention.

Multiple imputations will be adopted to manage all the missing data.

### Ethical considerations

Ethical approval for the study has been obtained from the Ethics Review Committee of The HK Polytechnic University. Informed proxy consents will be sought from the legal guardians or next of kin of all participants. Information sheets describing and explaining the nature of this study will be provided to the participants and their relatives. We will assure the participants that there will be no penalties if they withdraw from the study at any time. Anonymity and confidentiality will also be strictly protected. The staff from the long-term care homes and all RAs will be instructed to be careful during any procedures that may cause pain or discomfort to the participants (such as during the standardized exercise programme). All procedures should stop immediately if participants exhibit behavior that is resistant to the care or exercise. We will then continue to monitor participants’ vital signs and behavioral responses. Pain medications or non-pharmacological pain-relieving interventions may be provided according to the Protocol for participants in the experimental group, or usual pain management strategies for participants in the control group. If the situation becomes persistent or worse, we will consult the physiotherapist and the care home’s physician to manage participants’ pain or discomfort.

## Discussion

For decades, pain has been viewed as the fifth vital sign to be assessed, treated, and documented on a regular basis. People who suffer from pain problems should receive proper pain assessment and effective treatment. This is a fundamental human right [41,42]. Therefore, regular pain assessment and proper pain treatment are necessary in surgical units, intensive care units, medical units, and so on.

Despite the high prevalence of pain among older care home residents, pain management for this group remains suboptimal, especially for those with dementia. Lack of a standardized pain assessment tool and proper documentation are major barriers to successful pain management for residents with dementia [5]. Poor pain management in this population has been partly attributed to difficulties with efficient pain assessment. As the deterioration of cognition affects individuals’ ability to report pain, there is an increasing risk that pain will go undetected, leading to poor pain management. Unrelieved pain in residents with dementia may lead to fear of movement, resistance to care, anxiety, and agitated behavior [6]. Untreated pain not only affects their quality of life, but also causes more stress to caregivers dealing with problematic behavior.

One challenge for those caring for residents with dementia is difficulty in communication due to language deficits. Certain behaviors expressed by dementia sufferers serve as their communication [43,44]. This means that behaviors are expressions of their goals, needs, discomforts, and unmet needs, including feelings of pain and requests for pain treatment [45]. There is ample evidence showing that caregivers familiar with particular residents can detect pain based on observing their behavior [46]. However, this process of pain identification seems based mainly on intuition, and generally appears in an *ad hoc* manner. These are the major reasons why this intuitive pain identification lacks recognition by other professionals. It is suggested by pain experts that a systematic and consistent method of observing pain-related behavior in older people with dementia will improve the possibility of decoding the meanings behind expressed behaviors and increasing the recognition and treatment of pain/other unmet needs.

The most important aspect of the Protocol is its ability to alert caregivers to the benefits of pain treatment for older people with dementia. This study will improve pain management strategies for older residents with dementia. It will also illustrate the importance of regular systematic observational pain assessment. Through the incorporation of this Protocol, frontline healthcare providers can be more sensitive to the behavior of residents with dementia and can recognize those behaviors as symptoms of pain or other unmet needs. They can then increase their efforts to treat the underlying causes of pain. Perhaps the needs of individuals with dementia can be better met, tensions reduced, problematic behaviors decreased, and quality of life enhanced.

Although a similarly structured pain management protocol has been suggested previously [15,16], the recommendations were based on experts’ opinions rather than evaluation in research studies. The feasibility and effectiveness of this kind of pain management protocol, tailored to older people with dementia, will remain unknown until it has been implemented and evaluated in a clinical setting like this proposed scientific research study method. In view of the positive preliminary findings obtained in our pilot study [18], this Protocol should be evaluated in a randomized controlled trial with a larger sample size, so as to collect stronger evidence to demonstrate its effectiveness in pain assessment and management for non-communicative older care home residents. The findings will offer strong evidence that better strategies for pain management should be used in the care home daily routine.

## Trial status

The present study is currently recruiting care homes.

## Abbreviations

C-PAINAD: Chinese - pain assessment IN advanced dementia; C-MMSE: Cantonese-mini mental state examination; ICC: Interclass correlation coefficient; interRAI-HC: interRAI-Home Care; HK: Hong Kong; JL: Justina Yat Wa Liu (the first author and principal investigator of this trial); M-FVFT: Modified fuld verbal fluency test; MQS: Medication quantification scale; RA: Research assistant; SD: Standard deviation.

## Competing interests

The authors declare that they have no competing interests.

## Authors’ contributions

JYWL developed the original concept, drafted the Protocol, and wrote the final manuscript; CL provided technical advice and made critical revisions. Both authors read and approved the final manuscript.
